# In Vitro Glucuronidation of Wushanicaritin by Liver Microsomes, Intestine Microsomes and Expressed Human UDP-Glucuronosyltransferase Enzymes

**DOI:** 10.3390/ijms18091983

**Published:** 2017-09-19

**Authors:** Xiaodan Hong, Yuanru Zheng, Zifei Qin, Baojian Wu, Yi Dai, Hao Gao, Zhihong Yao, Frank J. Gonzalez, Xinsheng Yao

**Affiliations:** 1 College of Pharmacy, Jinan University, Guangzhou 510632, China; xiaodanhong0709@163.com (X.H.); c1394900674@163.com (Y.Z.); bj.wu@hotmail.com (B.W.); daiyi1004@163.com (Y.D.); tghao@jnu.edu.cn (H.G.); tyaoxs@jnu.edu.cn (X.Y.); 2 Guangdong Provincial Key Laboratory of Pharmacodynamic Constituents of TCM and New Drugs Research, College of Pharmacy, Jinan University, Guangzhou 510632, China; 3 Guangzhou Research and Creativity Biotechnology Co., Ltd., Guangzhou 510663, China; 4 Integrated Chinese and Western Medicine Postdoctoral research station, Jinan University, Guangzhou 510632, China; 5 Laboratory of Metabolism, Center for Cancer Research, National Cancer Institute, National Institutes of Health, Bethesda, MD 20892, USA; gonzalef@mail.nih.gov

**Keywords:** wushanicaritin, glucuronidation, UDP-glucuronosyltransferase, activity correlation analysis, relative activity factor, species difference

## Abstract

Wushanicaritin, a natural polyphenol compound, exerts many biological activities. This study aimed to characterize wushanicaritin glucuronidation by pooled human liver microsomes (HLM), human intestine microsomes and individual uridine diphosphate-glucuronosyltransferase (UGT) enzyme. Glucuronidation rates were determined by incubating wushanicaritin with uridine diphosphoglucuronic acid-supplemented microsomes. Kinetic parameters were derived by appropriate model fitting. Reaction phenotyping, the relative activity factor (RAF) and activity correlation analysis were performed to identify the main UGT isoforms. Wushanicaritin glucuronidation in HLM was efficient with a high *CL*_int_ (intrinsic clearance) value of 1.25 and 0.69 mL/min/mg for G1 and G2, respectively. UGT1A1 and 1A7 showed the highest activities with the intrinsic clearance (*CL*_int_) values of 1.16 and 0.38 mL/min/mg for G1 and G2, respectively. In addition, G1 was significantly correlated with β-estradiol glucuronidation (*r* = 0.847; *p* = 0.0005), while G2 was also correlated with chenodeoxycholic acid glucuronidation (*r* = 0.638, *p* = 0.026) in a bank of individual HLMs (*n* = 12). Based on the RAF approach, UGT1A1 contributed 51.2% for G1, and UGT1A3 contributed 26.0% for G2 in HLM. Moreover, glucuronidation of wushanicaritin by liver microsomes showed marked species difference. Taken together, UGT1A1, 1A3, 1A7, 1A8, 1A9 and 2B7 were identified as the main UGT contributors responsible for wushanicaritin glucuronidation.

## 1. Introduction

Xenobiotic metabolism, a ubiquitous natural response to foreign drugs, elicits initiating signals for many pathophysiological events [1]. According to different drug-metabolizing enzyme systems, xenobiotic biotransformation reactions could be classified to Phase I and Phase II reactions [2,3]. Normally, Phase I reactions included oxidation, reduction and hydrolysis, and cytochrome P450s enzymes (CYPs) in liver microsomal were the major Phase I metabolic enzymes [4]. The metabolites introduced several reactive functional substituents (–OH, –NH_2_, –SH, –H_2_) in metabolic soft spots of the parent drug [5]. Meanwhile, the reactions may produce pharmacologically-active metabolites or toxic metabolites, all of which were very likely to cause variations in drug efficacy [6]. Unlike the Phase I reactions, Phase II reactions always involve combination reactions. The major conjugation reactions were glucuronidation, acetylation, sulfation and methylation. Correspondingly, uridine diphosphate-glucuronosyltransferase (UGT), acetylase, sulfate transferase and methylase were responsible for Phase II catalytic reactions [7,8]. Actually, Phase II reactions were considered as one of the most important detoxification processes due to the obvious increased hydrophilicity of the conjugated metabolites, which could easily promote the excretion of the drug from the body [9,10].

It is well known that glucuronidation is the principal Phase II metabolism because it accounted for the clearance of ~35% drugs metabolized by Phase II enzymes [11]. In human, UDP-glucuronosyltransferases (UGTs) are a superfamily of enzymes involved in the glucuronidation of many endogenous and exogenous compounds (e.g., bilirubin, estradiol and polyphenols), especially in many clinical drugs [12,13]. The reaction involves the transfer of glucuronic acid from UDP-glucuronic acid (UDPGA) to an acceptor substrate to generate more polar and excretable compounds. Human UGT enzymes are classified into families UGT1, UGT2, UGT3 and UGT8 and subdivided into UGT1A, 2A and 2B subfamilies based on gene structure and sequence homology [14]. Traditionally, UGT1A (including nine members) and UGT2B (including seven members) were primarily responsible for the metabolism of xenobiotics or drugs [15]. Usually, the UGT1A and 2B enzymes contributed significantly to metabolism and detoxification of xenobiotics. UGT1A1, 1A3, 1A4, 1A5, 1A6 and 1A9 are expressed in the liver, the major site of glucuronidation, whereas UGT1A7, 1A8 and 1A10 are extrahepatic. All seven UGT2B enzymes (UGT2B4, 2B7, 2B10, 2B11, 2B15, 2B17 and 2B28) are found in the liver [16].

Wushanicaritin is a major active compound isolated from the traditional Chinese medicine *Epimedium* plants. Previous diphenyl picryl hydrazinyl radical (DPPH) radical scavenging activity tests indicated that wushanicaritin (IC_50_ = 35.3 μM) exhibited significant antioxidant activity comparable to vitamin C (IC_50_ = 32.0 μM) [17]. In addition, it displayed anti-inflammatory potential in murine macrophage cell lines, as well as in a mouse model of inflammation [18]. Similarly, the anti-inflammatory property of wushanicaritin in human immune cells, especially in monocytes, proved to be mediated, at least partially, via inhibition of the cluster of differentiation 14/toll-like receptor 4 (CD14/TLR4) signaling pathway [19]. Recently, it had been reported that the combination of wushanicaritin and the antiviral drug ganciclovir (GCV) is more effective in inducing extranodal NK/T-cell lymphoma (ENKL) cells’ apoptosis than wushanicaritin or GCV alone, which indicated that wushanicaritin exert significant antitumor effects [20].

So far, the metabolic pathways of wushanicaritin remain unknown. The presence of phenolic functional groups suggested that wushanicaritin may undergo glucuronidation. This knowledge is of great importance for a better understanding of wushanicaritin disposition and its mechanisms of action in vivo. In this study, we aim to characterize the metabolism of wushanicaritin via the glucuronidation pathway and to identify the main UGT enzymes involved in wushanicaritin glucuronidation. The rates of glucuronidation were determined by incubating wushanicaritin with uridine diphosphoglucuronic acid (UDPGA)-supplemented microsomes. Kinetic parameters were derived by fitting an appropriate model to the data. Several series of independent experiments including reaction phenotyping, determination of the relative activity factors (RAF) and activity correlation analyses were performed to identify the main UGT enzymes contributing to the hepatic metabolism of wushanicaritin. It was shown for the first time that wushanicaritin was efficiently metabolized via glucuronidation. Furthermore, UGT1A1, 1A3, 1A7, 1A8, 1A9 and 2B7 were identified as the main contributors to the glucuronidation of wushanicaritin.

## 2. Results

### 2.1. Structural Identification of Wushanicaritin Metabolites

**Figure 1:**
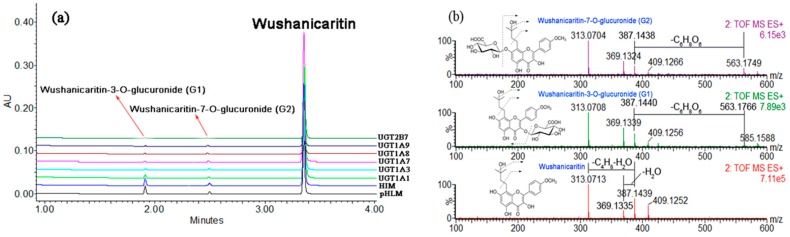
Ultra-high performance liquid chromatography analysis (**a**) and MS/MS spectrum (**b**) of wushanicaritin, wushanicaritin-3-*O*-glucuronide (G1) and wushanicaritin-7-*O*-glucuronide (G2). pHLM: pooled human liver microsomes; HIM: human intestine microsomes.

After incubation of wushanicaritin with uridine diphosphoglucuronic acid (UDPGA)-supplemented human liver microsomes (HLM), two additional peaks (*t*_R_ = 1.93 and 2.53 min), which have a similar ultraviolet (UV) absorption profile, in addition to wushanicaritin were detected by ultra-performance liquid chromatography coupled diode-array detector (UPLC-DAD) analysis (Figure 1a). Wushanicaritin exhibited a typical [M + H]^+^ ion at *m*/*z* 387.1439 and two main daughter ions at *m*/*z* 369.1335 and 313.0713 produced by losing a neutral fragment of H_2_O and C_4_H_8_, respectively (Figure 1b). For the metabolites, G1 and G2 had the same [M + H]^+^ ion at *m*/*z* 563.1749, which was 176.0325 Da higher than that of wushanicaritin (Figure 1b). Based on these data, they were characterized as mono-glucuronides of wushanicaritin.

In addition, to determine the exact substitution position of glucuronidation, G1 (about 1 mg) and G2 (less than 1 mg) were biosynthesized and purified by using pooled rat liver microsomes (RLM) and then structurally analyzed by ^1^H and ^13^C nuclear Magnetic Resonance (NMR) on a Bruker AV-600 spectrometer (Bruker, Newark, Germany). The signals for the ^1^H and ^13^C (600 MHz, DMSO-*d*_6_, 298.2 °C) of wushanicaritin and its two glucuronides were assigned and compared with those of wushanicaritin (Table S1). Meanwhile, their corresponding NMR parameters are shown in Table S2. Similarly, their ^1^H-NMR and ^13^C-NMR data are shown in Figures S1 and S2, respectively. The phenolic proton signals of the C-3 phenolic group in the ^1^H-NMR spectrum of G1 and the C-7 phenolic group in the ^1^H-NMR spectrum of G2 both disappeared, respectively. This evidence above indicated that the presence of glucuronidation substitutions should be at the C-3 and C-7 phenolic groups. Furthermore, the C-3 signal in the ^13^C-NMR spectrum of G1 shifted upfield to δ 135.0 (Table S1) caused by the glucuronidation of the C-3 phenolic group, while C-2 and C-4 shifted downfield to δ 155.4 and 177.7 (Table S1), respectively. However, the amount of G2 was less than 1 mg, and the ^13^ C-NMR data was not completely obtained. Taken altogether, G1 and G2 were identified as wushanicaritin-3-*O*-glucuronide (G1) and wushanicaritin-7-*O*-glucuronide (G2), respectively. On the other hand, these result were the same as previous studies [21,22]: wushanicaritin-3-*O*-glucuronide and wushanicaritin-7-*O*-glucuronide were eluted successively on a reverse C18 chromatographic column.

### 2.2. Glucuronidation of Wushanicaritin in Human Liver Microsomes (HLM) and Human Intestine Microsomes (HIM)

**Figure 2:**
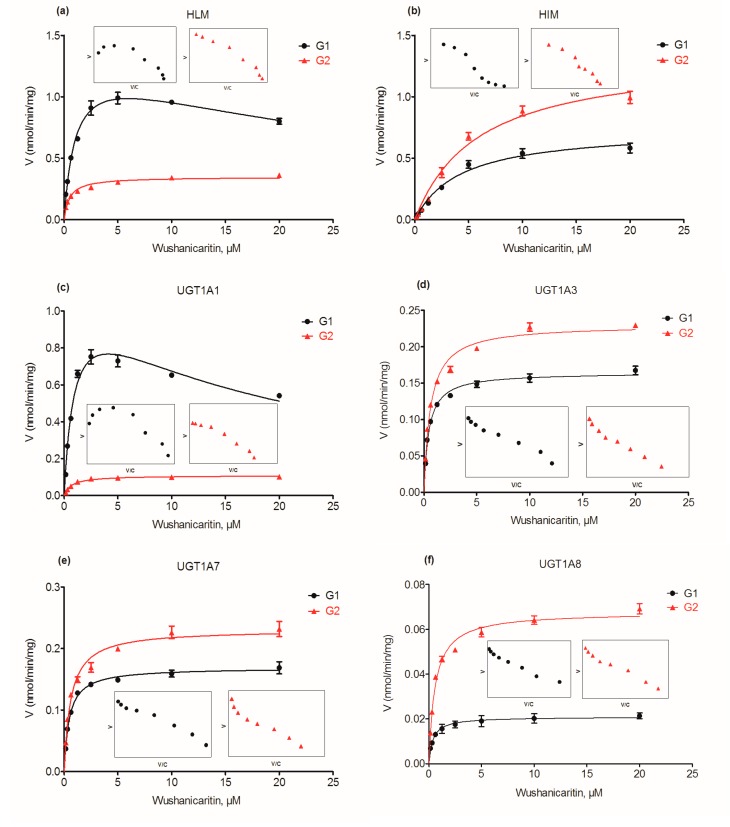

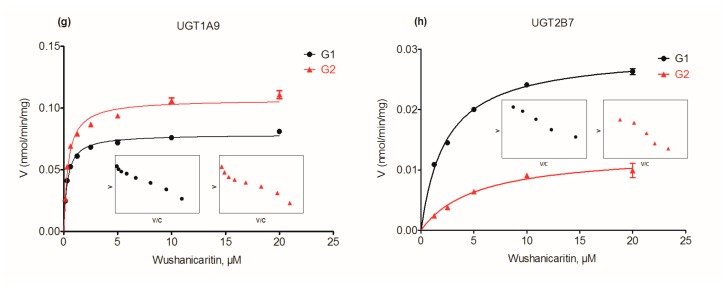
Kinetic profiles for glucuronidation of wushanicaritin by various types of microsomes. (**a**) Pooled human liver microsomes (HLM); (**b**) pooled human intestine microsomes (HIM); (**c**) expressed UGT1A1; (**d**) expressed UGT1A3; (**e**) expressed UGT1A7; (**f**) expressed UGT1A8; (**g**) expressed UGT1A9; (**h**) expressed UGT2B7; in each panel, the insert figure shows the corresponding Eadie–Hofstee plot. All experiments were performed in triplicate. Data are expressed as the mean ± SD. G1: wushanicaritin-3-*O*-glucuronide; G2: wushanicaritin-7-*O*-glucuronide.

Kinetic profiling revealed that the formation of wushanicaritin-3-*O*-glucuronide (G1) in HLM was well modeled by the substrate inhibition equation (Figure 2a), whereas wushanicaritin-7-*O*-glucuronide (G2) followed the classical Michaelis–Menten kinetics, as well as G1 and G2 in human intestine microsomes (HIM) (Figure 2b). The glucuronide formation of G1 and G2 in HLM was 1.34 and 0.35 nmol/min/mg, respectively. Similarly, the glucuronide formation of G1 and G2 in HIM was 0.74 and 1.34 nmol/min/mg, respectively. Glucuronidation G1 of wushanicaritin in HLM was efficient (*CL*_int_ = 1.25 mL/min/mg), following the substrate inhibition kinetics with *K*_m_ values of 1.07 μM. Glucuronidation G2 of wushanicaritin in HLM had a *CL*_int_ value of 0.69 mL/min/mg. Similarly, the *CL*_int_ values of G1 and G2 in HIM were 0.18 and 0.23 mL/min/mg, respectively, whereas the *K*_m_ values of G1 and G2 in HIM in the Michaelis–Menten model were 4.24 and 5.91 μM, respectively. In addition, the *K*_i_ values of G1 in HLM were 32.99 μM. The detailed parameters of G1 and G2 are listed in Table 1.

### 2.3. Reaction Phenotyping with Expressed UDP-Glucuronosyltransferase (UGT) Enzymes

**Figure 3:**
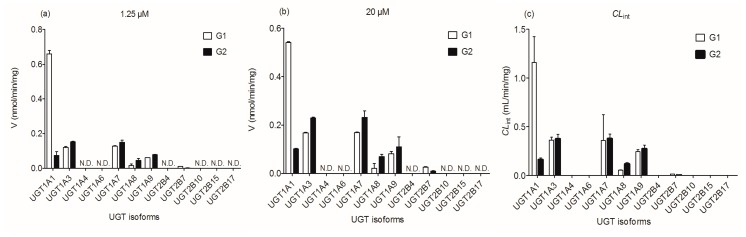
Comparisons of glucuronidation rates (**a**) 1.25 μM; (**b**) 20 μM and the intrinsic values (*CL*_int_) (**c**) of wushanicaritin by twelve expressed UGT enzymes. All experiments were performed in triplicate. Data are expressed as the mean ± SD. N.D.: not detected. G1: wushanicaritin-3-*O*-glucuronide; G2: wushanicaritin-7-*O*-glucuronide.

To identify the recombinant UGT enzymes involved in the glucuronidation of wushanicaritin, twelve expressed UGT enzymes were analyzed for their catalysis activities (expressed as nmol/min/mg protein) at the substrate concentrations of 1.25 μM (Figure 3a) and 20 μM (Figure 3b). The metabolic profiles for G1 and G2 were similar under two test substrate concentrations. All experiments were performed in triplicate. The formation of G1 and G2 mainly contributed to expression of UGT1A1, 1A3, 1A7, 1A8, 1A9 and 2B7 enzymes. The other seven UGT enzymes were not capable of the production toward G1 and G2. In general, UGT1A1 showed the highest glucuronidation activities for the formation of G1, while UGT1A7 was mainly responsible for the production of G2.

### 2.4. Glucuronidation Kinetics by Recombinant UGT Enzymes

Based on reaction phenotyping results, glucuronidation kinetics of active recombinant UGT enzymes were analyzed using a series of substrate concentrations. Obviously, the kinetic profiles of G1 by UGT1A1 were well modeled by the substrate inhibition equation (Figure 2c), which was in line with its glucuronidation profiles in HLM (Figure 2a), suggesting that UGT1A1 was indeed the most crucial enzyme responsible for hepatic glucuronidation of G1. Except UGT1A1, G1 glucuronidation of wushanicaritin by UGT1A3 (Figure 2d), 1A9 (Figure 2g) and 2B7 (Figure 2h) enzymes was all well modeled by Michaelis–Menten kinetics, which did not always followed the same kinetics as HLM. For the metabolite G1, the intrinsic clearance (*CL*_int_) values by UGT1A1, 1A3, 1A9 and 2B7 were 1.16, 0.36, 0.24 and 0.01 mL/min/mg, respectively (Table 1). On the contrary, the profiles of G2 by UGT1A1 (Figure 2c), 1A3 (Figure 2d), 1A9 (Figure 2g) and 2B7 (Figure 2h) were obviously modeled by the Michaelis–Menten kinetics, which were all the same as the glucuronidation profiles in HLM (Figure 2a). For G2, *CL*_int_ values by UGT1A1, 1A3, 1A9 and 2B7 were 0.16, 0.38, 0.27 and 0.002 mL/min/mg, respectively (Table 1). Hence, UGT1A1, 1A3, 1A9 and 2B7 were the main hepatic UGT enzymes for glucuronidation (G1 and G2) of wushanicaritin (Figure 3c).

On the other hand, HIM-mediated glucuronidation of G1 and G2 both well followed the Michaelis–Menten kinetics (Figure 2b). Similarly, UGT1A7 (Figure 2e) and 1A8 (Figure 2f) enzymes were both analyzed by the classical Michaelis–Menten kinetics. The *CL*_int_ values of G1 by UGT1A7 and 1A8 enzymes were 0.36 and 0.05 mL/min/mg, respectively (Table 1), while UGT1A7- and 1A8-mediated glucuronidation of G2 had *CL*_int_ values of 0.38 and 0.12 mL/min/mg, respectively (Table 1). These results indicated that gastrointestinal enzymes (UGT1A7 and 1A8) also played the most important roles in the glucuronidation of wushanicaritin (Figure 3c). Taken together, their intrinsic clearance (*CL*_int_) comparison plot is exhibited in Figure 3c. Kinetic profiles of UGT1A1, 1A3, 1A7, 1A8, 1A9 and 2B7 are shown in Figure 2c–h, whereas the calculated kinetic parameters are displayed in Table 1.

### 2.5. Contribution of UGT1A1, 1A3, 1A9 and 2B7 to Wushanicaritin Glucuronidation in HLM

**Figure 4:**
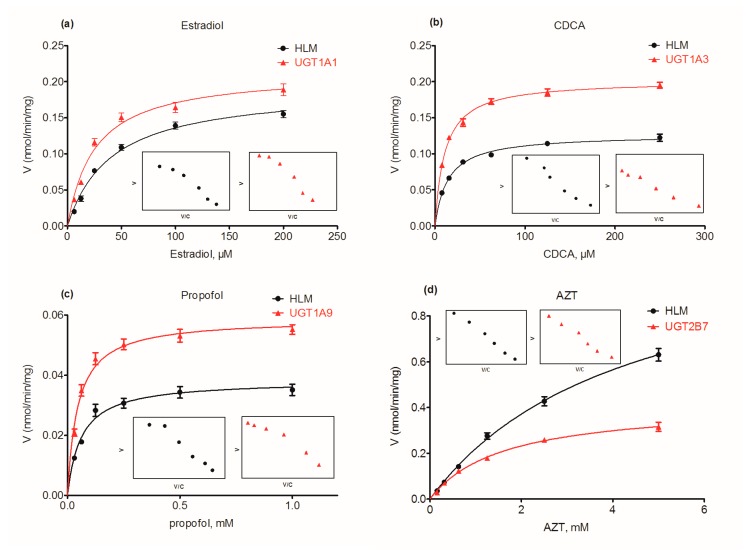
Kinetic profiles for β-estradiol (**a**), chenodeoxycholic acid (CDCA) (**b**), propofol (**c**) and zidovudine (AZT) (**d**) glucuronidation by pooled human liver microsomes (HLM) and individual UGTs enzymes; in each panel, the insert figure shows the corresponding Eadie–Hofstee plot. All experiments were performed in triplicate. Data are expressed as the mean ± SD.

To estimate the exact contribution of UGT1A1, 1A3, 1A9 and 2B7 to wushanicaritin glucuronidation in HLM, the RAF approach was calculated by the intrinsic clearance (*CL*_int_) values of β-estradiol, chenodeoxycholic acid (CDCA), propofol and zidovudine (AZT) glucuronidation in HLM and relative individual expressed UGT enzyme, respectively. The analytical conditions were adopted based on previous studies [12,23]. As a result, the kinetic profiles of β-estradiol, CDCA, propofol and AZT glucuronidation were modeled by the classical Michaelis–Menten kinetics (Figure 4). The derived RAFs for UGT1A1, 1A3, 1A9 and 2B7 were 0.55, 0.48, 0.49 and 1.04, respectively (Table 2). The scaled *CL*_int_ values of G1 and G2 were 0.642 (=1.16 × 0.0.55) mL/min/mg and 0.09 (=0.16 × 0.55) mL/min/mg for UGT1A1, which represented 51.2% and 12.9% of the *CL*_int_ values (1.25 and 0.69 mL/min/mg) in HLM. The scaled *CL*_int_ values of G1 and G2 were 0.17 (= 0.36 × 0.48) mL/min/mg and 0.18 (=0.38 × 0.48) mL/min/mg for UGT1A3, which represented 13.8% and 26.0% of the total glucuronidation activity in HLM. The scaled *CL*_int_ values of G1 and G2 were 0.12 (=0.24 × 0.49) mL/min/mg and 0.13 (=0.27 × 0.49) mL/min/mg for UGT1A9, which was 9.6% and 19.4% of total glucuronidation activity in HLM. The scaled *CL*_int_ values of G1 and G2 were 0.01 (=0.01 × 1.04) mL/min/mg and 0.002 (=0.002 × 1.04) mL/min/mg for UGT2B7, which was 1.1% and 0.3% of the total glucuronidation activity in HLM.

### 2.6. Activity Correlation Analysis

**Figure 5:**
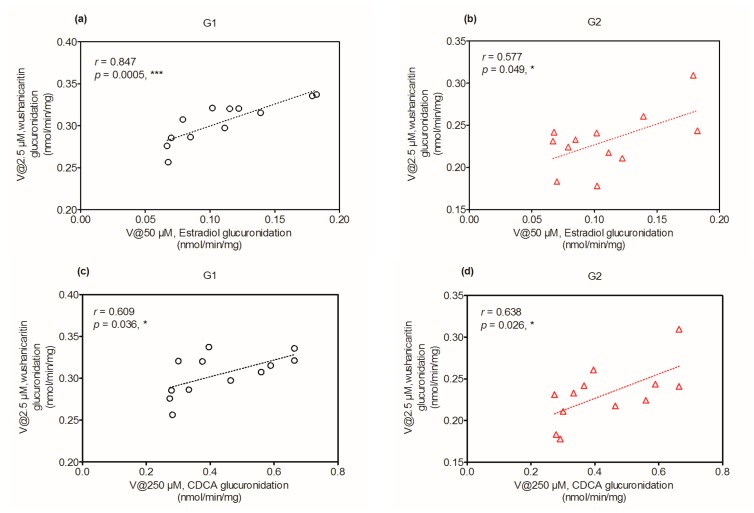

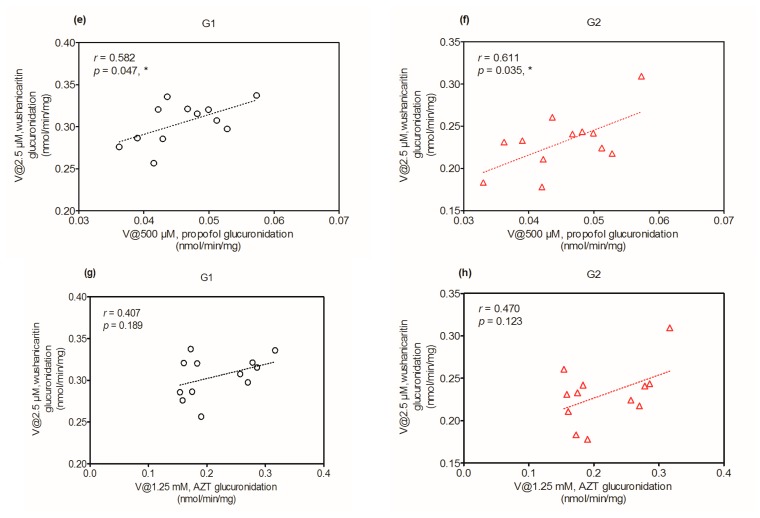
Correlation analysis between wushanicaritin 3-*O*-glucuronidation and β-estradiol glucuronidation (**a**), wushanicaritin 7-*O*-glucuronidation and β-estradiol glucuronidation (**b**) in a bank of individual human liver microsomes (*n* = 12); wushanicaritin 3-*O*-glucuronidation and CDCA glucuronidation (**c**), wushanicaritin 7-*O*-glucuronidation and CDCA glucuronidation (**d**) in a bank of individual human liver microsomes (*n* = 12); correlation analysis between wushanicaritin 3-*O*-glucuronidation and propofol glucuronidation (**e**), wushanicaritin 7-*O*-glucuronidation and propofol glucuronidation (**f**) in a bank of individual human liver microsomes (*n* = 12); correlation analysis between wushanicaritin 3-*O*-glucuronidation and AZT glucuronidation (**g**), wushanicaritin 7-*O*-glucuronidation and AZT glucuronidation (**h**) in a bank of individual human liver microsomes (*n* = 12). All experiments were performed in triplicate. CDCA: chenodeoxycholic acid; AZT: zidovudine. G1: wushanicaritin-3-*O*-glucuronide; G2: wushanicaritin-7-*O*-glucuronide.

As mentioned, glucuronidation activity of β-estradiol in HLM is a well-accepted functional marker for UGT1A1 [12]. Glucuronidation activities of individual HLMs (*n* = 12) toward wushanicaritin glucuronidation and β-estradiol glucuronidation were both determined. It was shown that wushanicaritin 3-*O*-glucuronidation (G1) and 7-*O*-glucuronidation (G2) were significantly correlated with β-estradiol glucuronidation with correlation factors (*r* = 0.847, *p* = 0.0005) and (*r* = 0.577, *p* = 0.049), respectively (Figure 5a,b). Similarly, G1 and G2 were significantly correlated with CDCA glucuronidation, (*r* = 0.609, *p* = 0.036) and (*r* = 0.638, *p* = 0.026), respectively (Figure 5c,d). Furthermore, wushanicaritin glucuronidation (G1 and G2) was strongly correlated with propofol glucuronidation, (*r* = 0.582, *p* = 0.047) and (*r* = 0.611, *p* = 0.035), respectively (Figure 5e,f). Moreover, G1 and G2 were also correlated with AZT glucuronidation (*r* = 0.407, *p* = 0.189) and (*r* = 0.470, *p* = 0.123), respectively (Figure 5g,h). The results indicated that UGT1A1, 1A3, 1A9 and 2B7 enzymes all played a critical role in wushanicaritin glucuronidation and were the main hepatic expressed UGTs for wushanicaritin glucuronidation.

### 2.7. Glucuronidation of Wushanicaritin by DLM, RLM, MkLM, RaLM and GpLM

**Figure 6:**
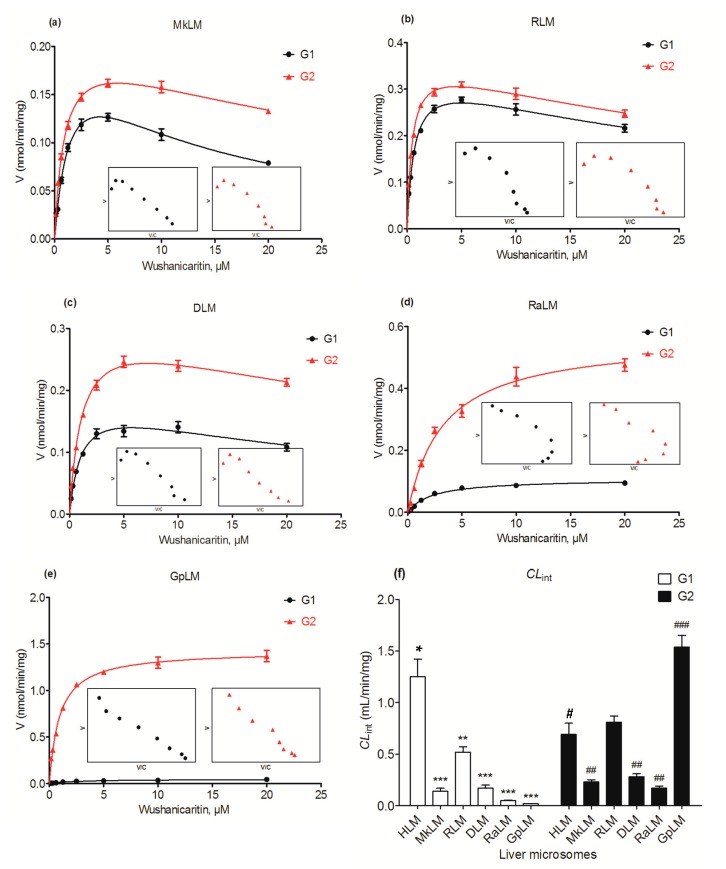
Kinetic profiles for the glucuronidation of wushanicaritin by various types of animal microsomes. (**a**) monkey liver microsomes (MkLM); (**b**) rat liver microsomes (RLM); (**c**) dog liver microsomes (DLM); (**d**) rabbit liver microsomes (RaLM); (**e**) guinea pig liver microsomes (GpLM); and the intrinsic clearance (*CL*_int_) values of human liver microsomes and five animals microsomes (**f**). In each panel, the insert figure shows the corresponding Eadie–Hofstee plot. All experiments were performed in triplicate. Data are expressed as the mean ± SD. HLM: human liver microsomes; G1: wushanicaritin-3-*O*-glucuronide; G2: wushanicaritin-7-*O*-glucuronide. * compared with the *CL*_int_ value of G1 in HLM, * *p* < 0.05, ** *p* < 0.01, *** *p* < 0.001; ^#^ compared with the *CL*_int_ value of G2 in HLM, ^#^ *p* < 0.05, ^##^ *p* < 0.01, ^###^ *p* < 0.001.

The apparent *V*_max_ and *K*_m_ values were determined for the formation of wushanicaritin glucuronides by animal microsomes (Table 1). Wushanicaritin was rapidly converted into glucuronides in human and five types of animal microsomes with high the intrinsic clearance (*CL*_int_) values of 0.02–1.25 mL/min/mg and 0.17–1.54 mL/min/mg for G1 and G2, respectively. The Michaelis–Menten model provided the best fit to the glucuronidation kinetics of wushanicaritin with guinea pig liver microsomes (GpLM) (Figure 6e). In contrast, wushanicaritin glucuronidation by monkey liver microsomes (MkLM) (Figure 6a), RLM (Figure 6b), dog liver microsomes (DLM) (Figure 6c) and rabbit liver microsomes (RaLM) (Figure 6d) all followed the substrate inhibition kinetics. The catalyzation efficiencies (reflected by *CL*_int_ values, Figure 6f) for G1 of human and animal microsomes followed the order of HLM (1.25 mL/min/mg) > RLM (0.52 mL/min/mg) > DLM (0.17 mL/min/mg) > MkLM (0.14 mL/min/mg) > RaLM (0.05 mL/min/mg) > GpLM (0.02 mL/min/mg). Similarly, the intrinsic clearance (*CL*_int_) values for G2 were GpLM (1.54 mL/min/mg) > RLM (0.81 mL/min/mg) > HLM (0.69 mL/min/mg) > DLM (0.28 mL/min/mg) > MkLM (0.23 mL/min/mg) > RaLM (0.17 mL/min/mg). Clearly, there was a marked species difference (up to 60.0-fold) in hepatic glucuronidation of wushanicaritin. Furthermore, rats were probably the best model for wushanicaritin glucuronidation studies in humans due to its appropriate kinetic parameters (Table 1).

## 3. Discussion

As a major bioactive compound in *Epimedium* plants, wushanicaritin has drawn much attention in the past decade. Modern pharmacological studies have clearly shown that wushanicaritin possesses diverse pharmacological activities, including antioxidant, anti-inflammatory and antitumor effects [17,18,19,20]. In contrast to the studies on pharmacological activity, the metabolic pathways and metabolic behavior of wushanicaritin have not been investigated. In this study, it was shown for the first time that wushanicaritin was efficiently metabolized in the liver via the glucuronidation pathway. In addition, this work provided strong evidence that UGT1A1, 1A3, 1A7, 1A8, 1A9 and 2B7 were the main contributors to glucuronidation of wushanicaritin. The results indicated that the Phase II metabolism glucuronidation was an important pathway for wushanicaritin clearance. Therefore, the role of UGT enzymes in determining the body exposure (bioavailability) and elimination of the compound should not be underestimated.

This study demonstrated that wushanicaritin could be rapidly glucuronidated in both HLM and HIM in the presence of UDPGA, while two mono-glucuronides were formed (Figure 1). The finding that UGT1A1, 1A3, 1A7, 1A8, 1A9 and 2B7 are the main enzymes contributing to the metabolism of capsaicin was strongly supported by three lines of evidence. First, of all hepatic UGT enzymes, UGT1A1, 1A3, 1A7, 1A8, 1A9 and 2B7 showed predominant activities towards wushanicaritin (Figure 3). Second, wushanicaritin glucuronidation was significantly correlated with β-estradiol glucuronidation (a functional marker of UGT1A1), CDCA glucuronidation (a functional marker of UGT1A3), propofol glucuronidation (a functional marker of UGT1A9) and AZT glucuronidation (a functional marker of UGT2B7) (Figure 5). Third, up to 75.7% (G1) and 58.6% (G2) of wushanicaritin glucuronidation in HLM were attributed to UGT1A1, 1A3, 1A9 and 2B7 based on the RAF approach. Although UGT1A7 and 1A8 also had high activities towards wushanicaritin (Figure 3c), the present study did not determine their roles in hepatic glucuronidation because they are gastrointestinal enzymes and hardly found in the liver [24,25].

The glucuronidation activity was obtained by kinetic profiling and modeling. Kinetic profiling required the determination of the rates of wushanicaritin glucuronidation at a series of wushanicaritin concentrations. The relative activities of different expressed UGT enzymes toward wushanicaritin glucuronidation were evaluated by the derived *CL*_int_ values. Use of *CL*_int_ (= *V*_max_/*K*_m_) as an indicator of UGTs enzyme activity was advantageous, because: (1) *CL*_int_ represents the catalytic efficiency of the enzyme and is independent of the substrate concentration; (2) compared with other kinetic parameters such as *K*_m_ and *V*_max_, *CL*_int_ is more relevant in the attempt to predict hepatic clearance in vivo [26]. Therefore, *CL*_int_ values were used to determine the wushanicaritin glucuronidation activity of different expressed UGTs in this study.

Characterization of wushanicaritin glucuronidation assumed a great role in the understanding of its pharmacokinetics and bioavailability. Oral bioavailability is a major factor in determining the biological actions of wushanicaritin in vivo. This study suggested that the oral bioavailability of wushanicaritin would be influenced by first-pass glucuronidation in the liver. Furthermore, UGT1A7 and 1A8 which were abundantly expressed in the intestine showed metabolic activities towards wushanicaritin (Figure 3c). Thus, it was highly possible that intestinal glucuronidation had an impact on the oral bioavailability. Moreover, the role of glucuronidation in determining the oral bioavailability of wushanicaritin should not be emphasized. On the other hand, UGT1A1, 1A3 and 1A9 are highly polymorphic enzymes [27,28]. As wushanicaritin was predominantly metabolized by these three enzyme, large pharmacokinetic variability was expected to exist among individuals with different UGT1A1, 1A3 and 1A9 genotypes. Considering that UGT1A1, 1A3 and 1A9 are the main contributing UGT isoforms in wushanicaritin glucuronidation, it is readily conceivable that wushanicaritin could be extensively glucuronidated after oral administration. Therefore, the activity and the excretion of these two wushanicaritin glucuronides should be further investigated.

Since metabolism is an important factor in determining the drug pharmacokinetics, toxicity and efficacy, it is critical to evaluate drug metabolism in the preclinical phases. A proper animal model appeared to be essential in the preclinical evaluation of drug metabolism. An acceptable animal model should bear close resemblance to humans in the metabolic pathways and metabolite formation kinetics [29]. In this study, comparative analysis of wushanicaritin glucuronidation in humans and five experimental animals were performed using liver microsomes. It was found that rat displayed similar kinetic parameters (within 2.5-fold variability) to humans (Table 1). The results suggested that rat might be a preferred choice to serve as a surrogate model for metabolism and pharmacokinetic studies of wushanicaritin.

Apart from the rapid glucuronidation of wushanicaritin in human liver and intestine, kidney may also participate in wushanicaritin glucuronidation in the human body. Taking into account the fact that UGT1A9 displayed a catalytic activity towards G1 and G2, it also could be abundantly expressed in kidney [30]. UGT1A9 may be the major contributor to the metabolic clearance of wushanicaritin in human kidney. Taken together, wushanicaritin could be metabolized by human UGTs and generates two wushanicaritin glucuronides. Kinetic characterization and activity correlation analysis assays demonstrated that UGT1A1, 1A3, 1A9 and 2B7 played important roles in hepatic glucuronidation of wushanicaritin, while UGT1A7 and 1A8 are two major contributors to the formation of two wushanicaritin glucuronides in HIM. In addition, *CL*_int_ values of wushanicaritin glucuronidation generated in HIM indicated a first-pass metabolism in the intestinal border after oral administration, and the following hepatic glucuronidation should lead to a rapid elimination of wushanicaritin from the human body. Moreover, UGT1A1, 1A3, 1A9 and 2B7 are highly polymorphic enzymes [27,28]. Since wushanicaritin was predominantly metabolized by these hepatic enzymes, a large pharmacokinetic variability was expected to exist among individuals with different UGT1A1, 1A3, 1A9 and 2B7 genotypes.

## 4. Materials and Methods

### 4.1. Materials

Uridine diphosphate glucuronic acid (UDPGA), magnesium chloride (MgCl_2_), alamethicin and d-saccharic-1,4-lactone were provided by Sigma-Aldrich (St. Louis, MO, USA). Pooled human liver microsomes (HLM), human intestinal microsomes (HIM), recombinant expressed human UGT Supersomes™ (UGT1A1, 1A3, 1A4, 1A6, 1A7, 1A8, 1A9, 2B4, 2B7, 2B10, 2B15 and 2B17), dog liver microsomes (DLM), rat liver microsomes (RLM), monkey liver microsomes (MkLM), rabbit liver microsomes (RaLM) and guinea pig liver microsomes (GpLM) were all obtained from Corning Biosciences (Corning, Corning, NY, USA). Wushanicaritin (purity > 98%) was purchased from Jingzhu Medical Technology Co., Ltd. (Nanjing, China). Wushanicaritin 3-*O*-glucuronidation (G1) and 7-*O*-glucuronidation (G2) were prepared according to the previous study [12]. The detailed NMR data of G1 and G2 are shown in Table S1, and their NMR spectra were displayed in Figures S1 and S2. β-estradiol, chenodeoxycholic acid (CDCA), propofol and zidovudine (AZT) were purchased from Aladdin Chemicals (Shanghai, China). All other chemicals and reagents were of analytical grade or the highest grade commercially available.

### 4.2. Glucuronidation Assay

Wushanicaritin was incubated with HLM, HIM and expressed UGTs enzymes to determine the rates of glucuronidation as published previously [12,23,31]. Briefly, the incubation mixture mainly contained 50 mM Tris-hydrochloric acid buffer (pH 7.4), 0.88 mM MgCl_2_, 22 µg/mL alamethicin, 4.4 mM saccharolactone and 3.5 mM UDPGA [32]. The reaction was terminated by adding ice-cold acetonitrile. The samples were vortexed and centrifuged at 13,800× *g* for 10 min. The supernatant was subjected to UPLC/Q-TOF-MS (Waters Corporation, Manchester, UK) analysis. Incubation without UDPGA served as the negative control to confirm the metabolites produced were UDPGA-dependent. Similarly, wushanicaritin was incubated with DLM, RLM, MkLM, RaLM and GpLM in the same way as for HLM. All experiments were performed in triplicate. Preliminary experiments were performed to ensure that the rates of glucuronidation were determined under linear conditions with respect to the incubation time and protein concentration.

### 4.3. Structural Identification of Wushanicaritin Glucuronide by UPLC/Q-TOF-MS

Metabolite screening of wushanicaritin glucuronide was performed using a UPLC-Q-TOF/MS system (Waters Corporation, Manchester, UK). Chromatographic separation was performed on a BEH C18 column (2.1 mm × 50 mm, 1.7 µm, Waters, Ireland, Part No. 186002350) guarded with a column temperature at 35 °C. The mobile phase consisted of water (A) and acetonitrile (B) (both including 0.1% formic acid, *V*/*V*) at a flow rate of 0.4 mL/min. The gradient elution program was 20% B from 0–0.5 min, 20–50% B from 0.5–3 min, 50–100% B from 3.0–3.5 min, maintaining 100% B from 3.5–4.0 min, 100–20% B from 4.0–4.5 min, keeping 20% B from 4.5–5.0 min.

The UPLC system was coupled to a hybrid quadrupole orthogonal time-of-flight (Q-TOF) tandem mass spectrometer (SYNAPT^TM^ G2 HDMS, Waters, Manchester, UK) with electrospray ionization (ESI). The operating parameters were as follows: capillary voltage, 3 kV (ESI+); sample cone voltage, 35 V; extraction cone voltage, 4 V; source temperature, 100 °C; desolvation temperature, 300 °C; cone gas flow, 50 L/h and desolvation gas flow, 800 L/h. The full scan mass range was 50–1500 Da. The method employed lock spray with leucine enkephalin (*m/z* 556.2771 in positive ion mode) to ensure mass accuracy.

### 4.4. Quantification of Wushanicaritin and Its Glucuronides

Due to the lack of a reference standard, quantification of wushanicaritin glucuronide was based on the standard curve of the parent compound (wushanicaritin) according to the assumption that the parent compound and its glucuronide have closely similar UV absorbance maxima [12,33]. A series of working solutions of wushanicaritin were determined by the Acquity™ UPLC I-Class system equipped with a BEH C18 column (2.1 mm × 50 mm, 1.7 µm, Waters, Ireland, Part No. 186002350). The gradient elution program was the same as the UPLC condition of the structural identification of wushanicaritin glucuronides above. The detection wavelength was set at 270 nm, and the injection volume was 8 µL.

The LOD and LOQ were calculated as 3-fold and 10-fold of the ratio of the signal-to-noise (*S/N*), respectively. The LOD and LOQ for wushanicaritin were 0.01 and 0.02 μM, respectively. Calibration curves were constructed by plotting wushanicaritin peak area ratios (Y) versus wushanicaritin concentrations (X) using a 1/*x*^2^ weighting factor. Acceptable linear correlation (Y = 13116X) was confirmed by correlation coefficients (*r*^2^) of 0.9998. The linear range was 0.02–50 μM. The accuracy and precision of the intra-day and inter-day error were both less than 2.9%.

### 4.5. Enzymes Kinetic Evaluation

Serial concentrations of wushanicaritin (0.156–20 µM) were incubated with HLM, HIM and expressed UGTs enzymes to determine wushanicaritin glucuronidation rates. The kinetic models of the Michaelis–Menten equation and substrate inhibition equation were fitted to the data of metabolic rates versus substrate concentrations and displayed in Equations (1) and (2), respectively. Appropriate models were selected by visual inspection of the Eadie–Hofstee plot [34]. Model fitting and parameter estimation were performed by GraphPad Prism V5 software (GraphPad Software, Inc., San Diego, CA, USA).

The parameter were as follow. *V* is the formation rate of the product. *V*_max_ is the maximal velocity. *K*_m_ is the Michaelis constant, and [*S*] is the substrate. *K*_si_ is the substrate inhibition constant. The intrinsic clearance (*CL*_int_) was derived by *V*_max_/*K*_m_ for the Michaelis–Menten and substrate inhibition models [12].

(1) $$V=\frac{V_{\max}\;\times [S]}{K_{m}+[S]}$$

(2) $$V=\frac{V_{\max}\;\times [S]}{K_{m}\;+\;[S]\left(1+\frac{\left[S\right]}{K_{si}}\right)}$$

### 4.6. Activity Correlation Analysis

According to the glucuronidation assay protocol as described previously [2,12], the metabolic activities of individual HLMs (*n* = 12) toward wushanicaritin, β-estradiol (a probe substrate for UGT1A1), CDCA (a probe substrate for UGT1A3), propofol (a probe substrate for UGT1A9) and zidovudine (a probe substrate for UGT2B7) were determined. Wushanicaritin (2.5 µM), β-estradiol (50 µM), CDCA (250 µM), propofol (500 µM) and zidovudine (1.25 mM) were separately incubated with UDPGA-supplemented individual HLM (1.0 mg/mL) for 120 min. Correlation analyses were performed between wushanicaritin glucuronidation (G1 and G2) and β-estradiol glucuronidation, wushanicaritin glucuronidation (G1 and G2) and CDCA glucuronidation, between wushanicaritin glucuronidation (G1 and G2) and propofol glucuronidation and between wushanicaritin glucuronidation (G1 and G2) and zidovudine glucuronidation. Correlation (Pearson) analyses were performed using GraphPad Prism V5 software (GraphPad Software, Inc., San Diego, CA, USA).

### 4.7. Contribution of UGT Isoforms

The contribution of individual expressed UGT enzymes to wushanicaritin glucuronidation in HLM was evaluated by the relative activity factor (RAF) approach as described in previous studies [2,12]. The relative activity factor was defined as the activity ratio of HLM to an expressed UGT enzyme (Supersome) toward a probe substrate for this enzyme using Equation (3). The relative amount of wushanicaritin glucuronidation in HLM attributed to an expressed UGT enzyme was estimated by multiplying the glucuronidation activity (i.e., the intrinsic clearance) derived with this enzyme by the corresponding RAF. The RAFs were derived for UGT1A1, 1A3, 1A9 and 2B7 using the well-recognized probe substrates β-estradiol, CDCA, propofol and AZT, respectively. The contributions of individual UGT enzyme were calculated according to Equation (4).

(3) $$\mathrm{RAF}=\frac{{CL}_{\mathrm{int}}\{\mathrm{probe},\mathrm{pHLM}\}}{{CL}_{\mathrm{int}}\{\mathrm{probe},\mathrm{Supersome}\}}$$

(4) $$\mathrm{Contribution}\;\mathrm{of}\;\mathrm{UGTs}=\frac{CL_{\mathrm{int}}(\mathrm{wushanica}\;\mathrm{ritin},\mathrm{UGTs})}{CL_{\mathrm{int}}(\mathrm{wushanica}\;\mathrm{ritin},\mathrm{pHLM})}\times \mathrm{RAF}$$

### 4.8. Statistical Analysis

Data are expressed as the mean ± SD (standard deviation). The mean differences between treatment and control groups were analyzed by Student’s *t*-test. The level of significance was set at *p* < 0.05 (*) or *p* < 0.01 (**) or *p* < 0.001 (***).

## 5. Conclusions

In conclusion, wushanicaritin 3-*O*-glucuronidation (G1) and wushanicaritin 7-*O*-glucuronidation (G2) were efficient in HLM with the intrinsic clearance (*CL*_int_) values of 1.25 and 0.69 mL/min/mg, respectively. Furthermore, they were determined in HIM with *CL*_int_ values of 0.18 and 0.23 mL/min/mg, respectively. In addition, UGT1A1, 1A3, 1A7, 1A8, 1A9 and 2B7 showed significant metabolic activities toward wushanicaritin. UGT1A1, 1A3, 1A9 and 2B7 were the main hepatic enzymes with the highest activities. Furthermore, G1 and G2 were both significantly correlated with β-estradiol glucuronidation, CDCA glucuronidation, propofol glucuronidation and AZT glucuronidation in a bank of individual HLMs (*n* = 12). On the basis of the RAF approach, it was estimated that UGT1A1, 1A3, 1A9 and 2B7, respectively, contributed 51.2%, 13.8%, 9.6% and 1.1% of wushanicaritin 3-*O*-glucuronidation in HLM. Similarly, UGT1A1, 1A3, 1A9 and 2B7 contributed 12.9%, 26.0%, 19.4% and 0.3% for wushanicaritin 7-*O*-glucuronidation in HLM, respectively. Moreover, the metabolic efficiency for G1 followed the order of human > rat > dog > monkey > rabbit > guinea pig. Similarly, the *CL*_int_ values for G2 were guinea pig > rat > human > dog > monkey > rabbit. Overall, UGT1A1, 1A3, 1A7, 1A8, 1A9 and 2B7 were the main contributors to the glucuronidation of wushanicaritin. Additionally, glucuronidation of wushanicaritin by liver microsomes showed a marked species difference.

## Figures and Tables

**Table 1:** Kinetic parameters of wushanicaritin glucuronidation by HLM, HIM and expressed UGT enzymes (mean ± SD). All experiments were performed in triplicate.

Protein Source	Metabolite	*V*_max_ (nmol/min/mg)	*K*_m_ or *S*_50_ (μM)	*K*_i_ (μM)	*CL*_int_ (mL/min/mg)	Model
HLM	G1	1.34 ± 0.08	1.07 ± 0.13	32.990 ± 6.717	1.25 ± 0.17	SI
	G2	0.35 ± 0.01	0.50 ± 0.08	N.A.	0.69 ± 0.11	MM
HIM	G1	0.74 ± 0.05	4.24 ± 0.75	N.A.	0.18 ± 0.03	MM
	G2	1.34 ± 0.10	5.91 ± 1.10	N.A.	0.23 ± 0.05	MM
UGT1A1	G1	1.13 ± 0.11	0.98 ± 0.20	17.150 ± 4.478	1.16 ± 0.27	SI
	G2	0.11 ± 0.003	0.68 ± 0.08	N.A.	0.16 ± 0.02	MM
UGT1A3	G1	0.17 ± 0.003	0.45 ± 0.04	N.A.	0.36 ± 0.03	MM
	G2	0.23 ± 0.007	0.61 ± 0.08	N.A.	0.38 ± 0.05	MM
UGT1A7	G1	0.17 ± 0.003	0.47 ± 0.03	N.A.	0.36 ± 0.03	MM
	G2	0.23 ± 0.007	0.61 ± 0.08	N.A.	0.38 ± 0.05	MM
UGT1A8	G1	0.02 ± 0.001	0.38 ± 0.03	N.A.	0.05 ± 0.004	MM
	G2	0.07 ± 0.002	0.58 ± 0.07	N.A.	0.12 ± 0.01	MM
UGT1A9	G1	0.08 ± 0.001	0.32 ± 0.03	N.A.	0.24 ± 0.02	MM
	G2	0.11 ± 0.003	0.39 ± 0.06	N.A.	0.27 ± 0.04	MM
UGT2B7	G1	0.03 ± 0.001	2.34 ± 0.19	N.A.	0.01 ± 0.001	MM
	G2	0.01 ± 0.001	5.28 ± 0.98	N.A.	0.002 ± 0.001	MM
MkLM	G1	0.22 ± 0.02	1.58 ± 0.25	11.57 ± 2.15	0.14 ± 0.03	SI
	G2	0.21 ± 0.008	0.94 ± 0.08	36.23 ± 5.13	0.23 ± 0.02	SI
RLM	G1	0.34 ± 0.01	0.66 ± 0.06	37.11 ± 5.82	0.52 ± 0.05	SI
	G2	0.37 ± 0.009	0.45 ± 0.03	44.22 ± 5.42	0.81 ± 0.06	SI
DLM	G1	0.19 ± 0.01	1.11 ± 0.16	29.09 ± 6.58	0.17 ± 0.03	SI
	G2	0.32 ± 0.02	1.17 ± 0.13	44.14 ± 9.09	0.28 ± 0.03	SI
RaLM	G1	0.11 ± 0.004	2.22 ± 0.26	N.A.	0.05 ± 0.006	MM
	G2	0.56 ± 0.02	3.29 ± 0.39	N.A.	0.17 ± 0.02	MM
GpLM	G1	0.05 ± 0.002	2.53 ± 0.30	N.A.	0.02 ± 0.002	MM
	G2	1.43 ± 0.06	0.93 ± 0.06	N.A.	1.54 ± 0.11	MM

Notes: SI, substrate inhibition model; MM, Michaelis–Menten model; N.A., not available. pHLM: pooled human liver microsomes; HIM: human intestine microsomes; MkLM: monkey liver microsomes; RLM: rat liver microsomes; DLM: dog liver microsomes; RaLM: rabbit liver microsomes; GpLM: guinea pig liver microsomes; G1: wushanicaritin-3-*O*-glucuronide; G2: wushanicaritin-7-*O*-glucuronide.

**Table 2:** Kinetic parameters and relative activity factor (RAF) values of substrate glucuronidation by pooled human liver microsomes (HLM) and individual expressed UGT enzyme (mean ± SD). All experiments were performed in triplicate.

Substrate	Protein Source	*V*_max_ (nmol/min/mg)	*K*_m_ (μM)	*CL*_int_ (μL/min/mg)	Model	RAF
β-estradiol	HLM	0.19 ± 0.09	40.96 ± 5.44	4.68 ± 0.66	MM	0.55
	UGT1A1	0.21 ± 0.01	25.31 ± 4.28	8.45 ± 1.50	MM	
CDCA	HLM	0.13 ± 0.003	14.32 ± 1.16	8.86 ± 0.74	MM	0.48
	UGT1A3	0.20 ± 0.003	10.90 ± 0.74	18.51 ± 1.28	MM	
Propofol	HLM	0.04 ± 0.001	60.71 ± 9.145	0.63 ± 0.10	MM	0.49
	UGT1A9	0.06 ± 0.002	45.91 ± 5.91	1.28 ± 0.17	MM	
AZT	HLM	1.18 ± 0.53	4359.0 ± 341.4	0.27 ± 0.02	MM	1.04
	UGT2B7	0.42 ± 0.01	1604.0 ± 121.0	0.26 ± 0.02	MM	

Notes: MM, Michaelis–Menten model. CDCA: chenodeoxycholic acid; AZT: zidovudine.

## Supplementary Materials

Supplementary materials can be found at www.mdpi.com/1422-0067/18/9/1983/s1.
