# Supplementary material for: In Vitro Glucuronidation of Wushanicaritin by Liver Microsomes, Intestine Microsomes and Expressed Human UDP-Glucuronosyltransferase Enzymes
Source: Int J Mol Sci. 2017 Sep 19;18(9):1983. doi: 10.3390/ijms18091983 (PMC5618632; doi:10.3390/ijms18091983)
Supplement: Supplementary file 1 [file ijms-18-01983-s001.pdf]

In Vitro Glucuronidation of Wushanicaritin by Liver Microsomes, Intestine Microsomes and Expressed Human UDP-Glucuronosyltransferase Enzymes

Xiaodan Hong, Yuanru Zheng, Zifei Qin, Baojian Wu, Yi Dai, Hao Gao, Zhihong Yao, Frank J Gonzalez and Xinsheng Yao

Table S1. ^{1}H-NMR and ^{13}C-NMR data for wushanicaritin, wushanicaritin-3-*O*-glucuronide (G1) and wushanicaritin-7-*O*-glucuronide (G2) (600 MHz, DMSO-d$_6$, 298.2 °C).

Assignment		wushanicaritin		wushanicaritin-3-*O*-glucuronide		wushanicaritin-7-*O*-glucuronide	
^{1}H	^{13}C	δ_H	δ_C	δ_H	δ_C	δ_H	δ_C
5-OH	C-5	12.34 (s, 1H)	158.1	12.34 (s, 1H)	158.1	12.34 (s, 1H)	n.a.
7-OH	C-7	10.62 (s, 1H)	161.4	10.63 (s, 1H)	161.2	—	n.a.
3-OH	C-3	9.48 (s, 1H)	135.9	—	135.0	9.44 (s, 1H)	n.a.
H-3'/H-5'	C-3'/C-5'	8.23 (d, 2H, *J* = 9.0 Hz)	114.0	8.25 (d, 2H, *J* = 8.4 Hz)	113.7	8.26 (d, 2H, *J* = 8.5 Hz)	n.a.
H-2'/H-6'	C-2'/C-6'	7.09 (d, 2H, *J* = 9.0 Hz)	129.3	7.04 (d, 2H, *J* = 8.5 Hz)	129.3	7.10 (d, 2H, *J* = 8.5 Hz)	n.a.
H-6	C-6	6.29 (s, 1H)	97.8	6.29 (s, 1H)	98.4	6.58 (s, 1H)	n.a.
13-OH	C-13	4.32 (s, 1H)	68.8	4.31 (s, 1H)	68.9	4.31 (s, 1H)	n.a.
4'-OCH_3	C-4'	3.85 (s, 3H)	55.4	3.85 (s, 3H)	55.4	3.85 (s, 3H)	n.a.
H-11	C-11	2.78 (m, 2H)	17.4	2.70 (m, 2H)	17.5	2.84 (m, 2H)	n.a.
H-12	C-12	1.56 (m, 2H)	42.9	1.53 (m, 2H)	43.0	1.62 (m, 2H)	n.a.
H-14/H-15	C-14/C-15	1.20 (s, 6H)	29.2	1.23 (s, 6H)	29.1	1.23 (s, 6H)	n.a.
	C-10	—	103.0	—	103.8	—	n.a.
	C-9	—	153.5	—	153.5	—	n.a.
	C-8	—	106.9	—	107.0	—	n.a.
	C-4	—	176.3	—	177.7	—	n.a.
	C-4′	—	160.5	—	160.4	—	n.a.
	C-2	—	146.0	—	155.4	—	n.a.
	C-1′	—	123.6	—	123.8	—	n.a.
G1		—	—	5.18 (d, 1H, *J* = 5.2 Hz)	104.0	5.17 (d, 1H, *J* = 5.5 Hz)	n.a.
G2		—	—	3.77 (m, 1H)	74.3	n.a.	n.a.
G3		—	—	3.55 (m, 1H)	77.1	n.a.	n.a.
G4		—	—	3.64 (m, 1H)	72.5	n.a.	n.a.
G5		—	—	3.88 (d, 1H, *J* = 8.5 Hz)	76.8	3.97 (d, 1H, *J* = 8.4 Hz)	n.a.
G6		—	—	—	164.7		n.a.

Note: n.a. means that the ^{1}H-NMR and ^{13}C-NMR data were not completely obtained due to a very low amount of wushanicaritin-7-*O*-glucuronide (G2).

Table S2. The corresponding NMR parameters of wushanicaritin, wushanicaritin-3-*O*-glucuronide and wushanicaritin-7-*O*-glucuronide.

No.	Parameters	Values					
		**Wushanicaritin**		**Wushanicaritin-3-*O*-glucuronide (G1)**		**Wushanicaritin-7-*O*-glucuronide (G2)**	
		δ_H	δ_C	δ_H	δ_C	δ_H	δ_C
1	Data File Name	C:/icaritin/NMR/ICT-386/1/fid	C:/icaritin/NMR/ICT-386/2/fid	C:/icaritin/NMR/ICT-386-G1/1/fid	C:/icaritin/NMR/ICT-386-G1/2/fid	C:/icaritin/NMR/ICT-386-G2/1/fid	C:/icaritin/NMR/ICT-386-G2/2/fid
2	Title	ICT-386.1.fid	ICT-386.2.fid	ICT-386-G1.1.fid	ICT-386-G1.2.fid	ICT-386-G2.1.fid	ICT-386-G2.2.fid
3	Comment	PROTON DMSO {D:\Data} qinzifei 21	C13CPD DMSO {D:\Data} qinzifei 21	PROTON DMSO {D:\Data} qinzifei 16	C13CPD DMSO {D:\Data} qinzifei 16	PROTON DMSO {D:\Data} qinzifei 17	C13CPD DMSO {D:\Data} qinzifei 17
4	Origin	Bruker BioSpin GmbH	Bruker BioSpin GmbH	Bruker BioSpin GmbH	Bruker BioSpin GmbH	Bruker BioSpin GmbH	Bruker BioSpin GmbH
5	Owner	nmrsu	nmrsu	nmrsu	nmrsu	nmrsu	nmrsu
6	Site						
7	Instrument	spect	spect	spect	spect	spect	spect
8	Author						
9	Solvent	DMSO	DMSO	DMSO	DMSO	DMSO	DMSO
10	Temperature	298.2	298.1	298.2	298.2	298.2	298.1
11	Pulse Sequence	zg30	zgpg30	zg30	zgpg30	zg30	zgpg30
12	Experiment	1D	1D	1D	1D	1D	1D
13	Probe	5 mm CPTCI 1H-13C/15N/D Z-GRD Z117768/0031	5 mm CPTCI 1H-13C/15N/D Z-GRD Z117768/0031	5 mm CPTCI 1H-13C/15N/D Z-GRD Z117768/0031	5 mm CPTCI 1H-13C/15N/D Z-GRD Z117768/0031	5 mm CPTCI 1H-13C/15N/D Z-GRD Z117768/0031	5 mm CPTCI 1H-13C/15N/D Z-GRD Z117768/0031
14	Number of Scans	32	200	64	501	64	1037
15	Receiver Gain	10.2	197.9	10.2	176.9	11.0	176.9
16	Relaxation Delay	1.0000	2.0000	1.0000	2.0000	1.0000	2.0000
17	Pulse Width	7.0500	11.8000	7.0500	11.8000	7.0500	11.8000
18	Presaturation Frequency						
19	Acquisition Time	3.4079	0.9044	3.4079	0.9044	3.4079	0.9044
20	Acquisition Date	2017-07-13T16:39:44	2017-07-14T00:12:14	2017-08-10T13:34:53	2017-08-10T10:32:57	2017-08-10T11:15:14	2017-08-10T11:19:58
21	Modification Date	2017-07-13T16:39:46	2017-07-14T00:12:16	2017-08-10T13:36:30	2017-08-10T10:52:58	2017-08-10T11:16:25	2017-08-10T12:09:08
22	Class						
23	Spectrometer Frequency	600.15	150.93	600.15	150.93	600.15	150.93
24	Spectral Width	9615.4	36231.9	9615.4	36231.9	9615.4	36231.9
25	Lowest Frequency	−126.5	−761.5	−126.5	−761.5	−126.5	−761.5
26	Nucleus	1H	13C	1H	13C	1H	13C
27	Acquired Size	32,768	32,768	32,768	32,768	32,768	32,768
28	Spectral Size	65,536	65,536	65,536	65,536	65,536	65,536

(A)

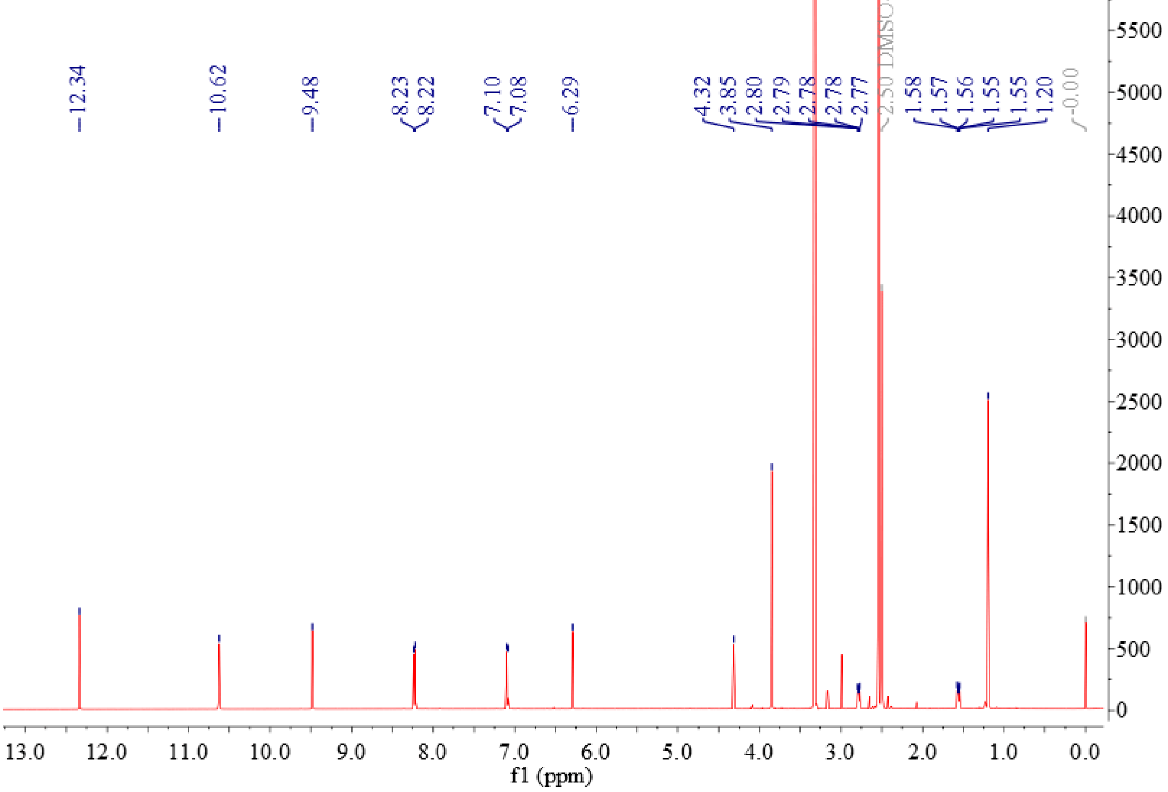

12.34
10.62
9.48
8.23
8.22
7.10
7.08
6.29
4.32
3.85
2.80
2.79
2.78
2.78
2.77
2.50 DMSO-d6
1.58
1.57
1.56
1.55
1.55
1.20
-0.00
5500
5000
4500
4000
3500
3000
2500
2000
1500
1000
500
0
13.0 12.0 11.0 10.0 9.0 8.0 7.0 6.0 5.0 4.0 3.0 2.0 1.0 0.0
f1 (ppm)

(B)

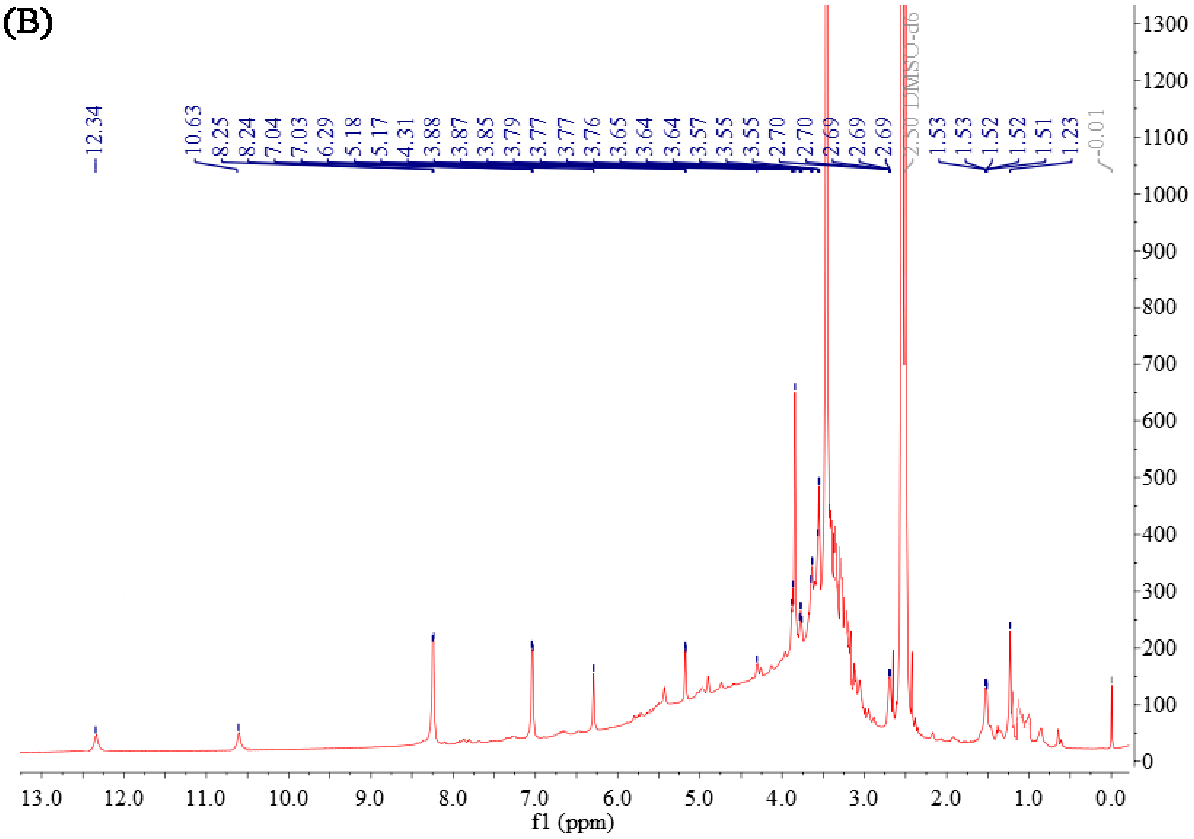

12.34
10.63
8.25
8.24
7.04
7.03
6.29
5.18
5.17
4.31
3.88
3.87
3.85
3.79
3.77
3.77
3.76
3.65
3.64
3.64
3.57
3.55
3.55
2.70
2.70
2.69
2.69
2.69
2.50 DMSO-d6
1.53
1.53
1.52
1.52
1.51
1.23
-0.01
1300
1200
1100
1000
900
800
700
600
500
400
300
200
100
0
13.0 12.0 11.0 10.0 9.0 8.0 7.0 6.0 5.0 4.0 3.0 2.0 1.0 0.0
f1 (ppm)

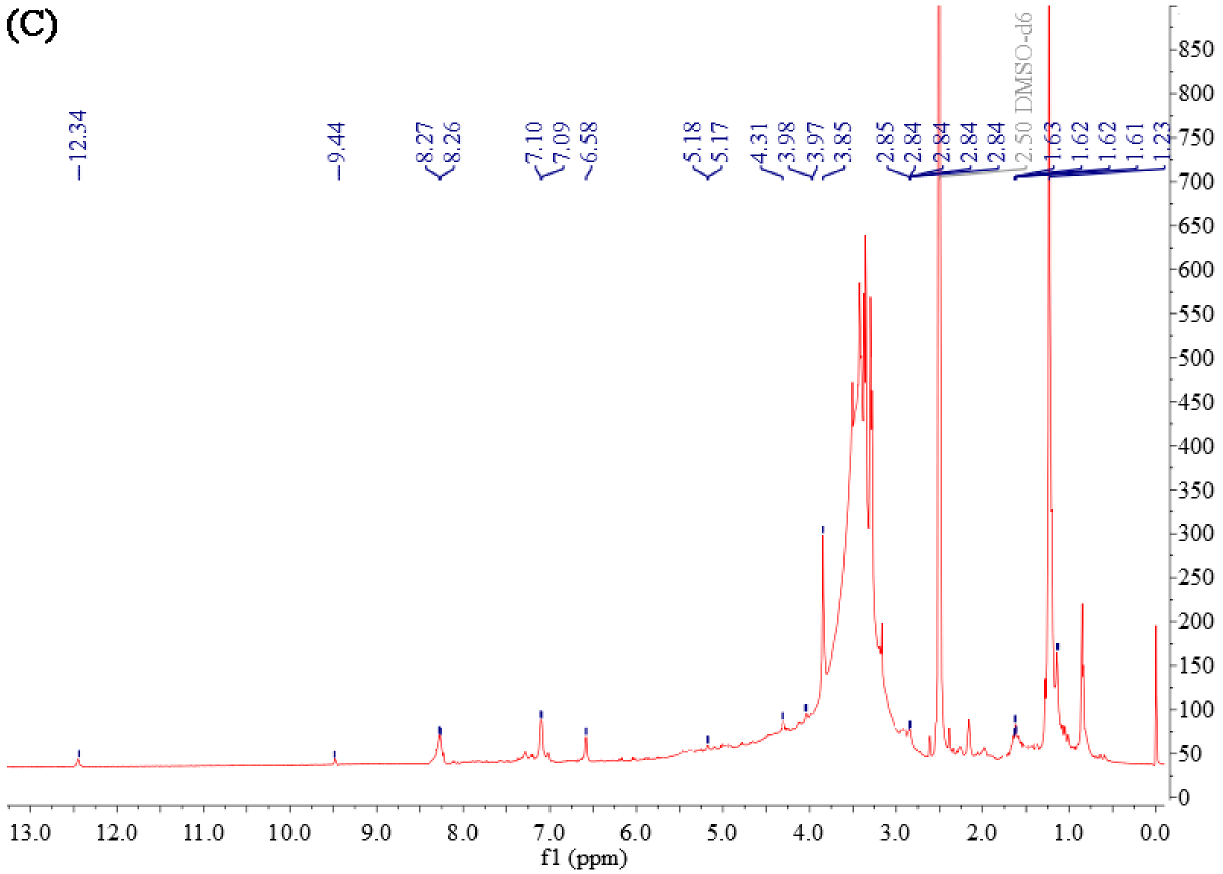

Figure S1. ^{1}H NMR spectrum of wushanicaritin (**A**), wushanicaritin-3-*O*-glucuronide (**B**) and wushanicaritin-7-*O*-glucuronide (**C**).

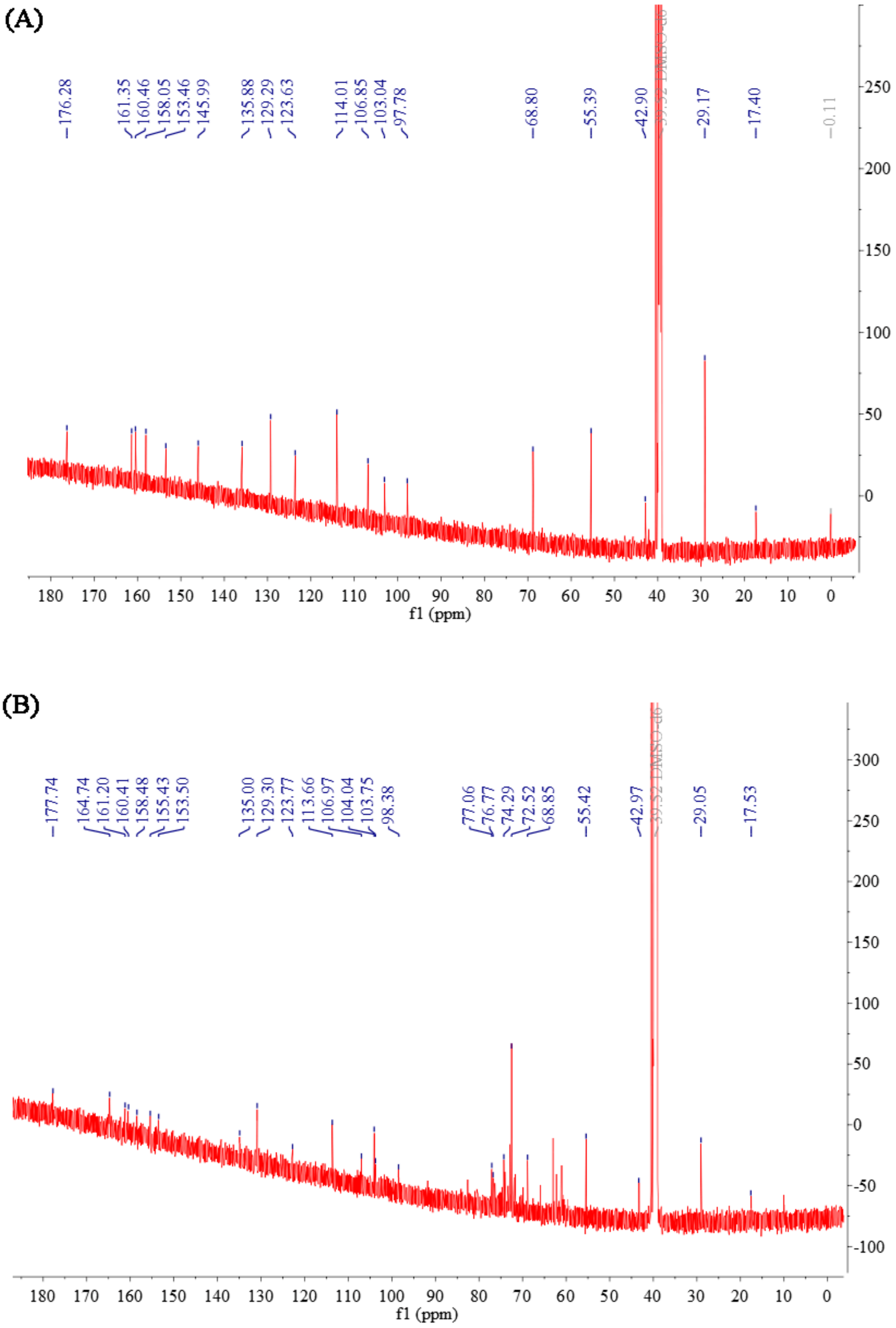

Figure S2. ^{13}C NMR spectrum of wushanicaritin (**A**) and wushanicaritin-3-*O*-glucuronide (**B**).
